# Supplementary material for: Predictors of bleeding complications during catHeter-dirEcted thrombolysis for peripheral arterial occlusions (POCHET)
Source: PLoS One. 2024 May 9;19(5):e0302830. doi: 10.1371/journal.pone.0302830 (PMC11081216; doi:10.1371/journal.pone.0302830)
Supplement: S1 File — (DOCX) [file pone.0302830.s001.docx]

**Supplementals**

**S1. Standard thrombolytic treatment of the lower limb**

1. Indication for thrombolytic treatment of the lower limb

Acute occlusion of an iliac or more distal native artery or venous bypass of the lower limb within six weeks of onset of ischemia.

2. Contraindications

Contraindications are subdivided into absolute and relative contraindications. In case of one or more present absolute contraindications, thrombolytic treatment is not feasible. If one or more relative contraindications is present, the possibility of thrombolytic treatment is up to the clinical judgement of the attending physician.

Absolute contraindications

- Severe limb ischemia for which operative treatment is indicated
- Recent stroke within three months
- Major surgery within 10 days
- Trauma within 10 days
- Puncture of any major artery within 10 days without closure device
- Epidural line in situ
- Active bleeding
- Clotting disorder
- Pregnancy
- Allergy to thrombolytic medication

Relative contraindications

- Operation or trauma beyond 10 days after inclusion
- Kidney and/or liver insufficiency
- Malignancy
- Severe hypertension
- Sepsis
- Ulcerative diseases of stomach or duodenum
- Vascular malformations and esophageal varices
- Allergy to radiologic contrast solutions
- Age above 80 years

3. Laboratory tests before thrombolytic treatment

Hemoglobin, hematocrit, leucocytes, thrombocytes, HbA1c, type and screen

Creatinine, eGFR, Sodium, Potassium, CRP, Glucose

Cholesterol, Triglycerides, LDL, HDL

PT, aPTT, INR, Fibrinogen, D-Dimer

4. Preparation and Angiography

Eating is prohibited 2 hours before intervention. Two peripheral venous catheters and a urinal tract catheter is inserted. The sheath is placed on the contralateral side to the occlusion, unless local status recommends otherwise. The artery will be punctured under ultrasound guidance and a 5-6Fr sheath is placed. If needed, a mechanical thrombectomy is performed with devices locally preferred. Finally an infusion catheter will be inserted.

5. Type of thrombolytic medication and dosage

Either alteplase or urokinase is used as thrombolytic medication. The intra-arterial dose is 1mg/h (=10ml/h) for alteplase and 100.000 IE/h for urokinase respectively. A bolus of the thrombolytic medication is given only when a mechanical thrombectomy is performed prior to the start of thrombolytic treatment. A bolus consists of 5mg alteplase or 250.000 IE or urokinase.

6. Heparin

As standard protocol, 5000 IU of heparin are administered intra-arterially after sheath placement. During thrombolytic treatment, 15IU/h of heparin is administered through a side branch of the sheath.

7. Patient monitoring

Patients receiving thrombolytic treatment will be monitored in at least a medium care facility and put to beds rest with a maximal upright elevation of 30 degrees. If necessary, turning is possible over the contra-lateral side of the sheath. During treatment, blood samples will be taken 4 times daily of at least haemoglobin levels. These blood samples will be taken from a peripheral venous catheter which will not be used for fluid or drug administration. Plasma fibrinogen levels will not be taken during fibrinolytic treatment. A new angiography is made at least every 24 hours.

8. Other anticoagulants during fibrinolytic treatment

Any anti-platelet medication, such as ascal, clopidogrel and dypiridamol or any combination can be given throughout the fibrinolytic treatment. Anti-thrombotic medication (e.g. NOAC’s, vitamin K antagonists, heparin or low-molecular weight heparins (LMWH’s) should be ceased during fibrinolytic treatment. Low dose LMWH’s to avoid deep venous thrombosis is not recommended.

9. End of treatment

The maximum time for fibrinolytic treatment is 72 hours. After the thrombolytic treatment has stopped, either because of the maximum time of 72h has been exceeded or when sufficient result has been achieved, a closure device is placed after sheath removal. If, after closure device placement, the groin is not dry instantaneous, manual compression is performed for several minutes. Before sheath removal, a bolus of heparin is not recommended. The patient is put to beds rest for at least three hours. The type of prescribed anti-thrombotic medication after fibrinolytic therapy is chosen by the treating physician.
