# Supplementary material for: Predictors of bleeding complications during catHeter-dirEcted thrombolysis for peripheral arterial occlusions (POCHET)
Source: PLoS One. 2024 May 9;19(5):e0302830. doi: 10.1371/journal.pone.0302830 (PMC11081216; doi:10.1371/journal.pone.0302830)
Supplement: S2 File — (DOCX) [file pone.0302830.s002.docx]

| Endpoint | Definition |
| --- | --- |
| **Primary Endpoint**: Non acces-site related major bleeding | Any of the following, not related to the access site for thrombolysis   - bleeding related death - intracranial bleeding - intra-organ bleeding - surgical - , radiological - or endoscopic intervention related to bleeding - necessity of inotropic medication |
| **Secondary endpoints** |  |
| non-major bleeding complications | Any bleeding leading to early cessation of thrombolysis, including access site bleeding |
| Other bleeding complications | Any bleeding complication not meeting the criteria for other bleeding complications |
| Trash | Disintegration of primary arterial occlusion leading to new occlusions more distally for which other treatment than ongoing thrombolysis or oral medication is needed. |
| Compartment syndrome | Compartment syndrome for which fasciotomy is needed |
| 30 day re-intervention | Any intervention within 30 days of the start of thrombolysis |
| 30 day major amputation | Above the ankle amputation within 30 days of thrombolysis |
| All cause mortality | Any cause of death |
