# Supplementary material for: Predictors of bleeding complications during catHeter-dirEcted thrombolysis for peripheral arterial occlusions (POCHET)
Source: PLoS One. 2024 May 9;19(5):e0302830. doi: 10.1371/journal.pone.0302830 (PMC11081216; doi:10.1371/journal.pone.0302830)
Supplement: S3 File — (DOCX) [file pone.0302830.s003.docx]

| Variable | Description |
| --- | --- |
| pochet_number | Study Number |
| UPID | Unique patient identifier |
| hospital | Hospital the patient was treated in |
| gender | Gender |
| age | Age at presentation |
| length | Height in cm |
| weight | Weight in Kg |
| BMI | BMI |
| date_ic | Date if Informed Consent |
| vg_ht | History of Hypertension |
| vg_hc | History of Hypercholesterolaemia |
| vg_dm | History of Diabetes Mellitus |
| vg_smoke | History of Smoking |
| vg_mi | History of myocardial infarction |
| vg_cabg_pci | History of CABG or PTCA/PCI |
| vg_af | History of Atrial Fibrillation |
| vg_cva | History of CVA |
| vg_malig | History of Malignancy |
| vg_maligtreat | Currently treated for malignancy |
| vg_copd | History of COPD |
| vg_paod | History of PAOD |
| vg_paod_pta | History of PTA treatment for PAOD |
| vg_paod_tea | History of TEA treatment for PAOD |
| vg_paod_bypass | History of Bypass treatment for PAOD |
| vg_paod_bypass_type | Type of bypass |
| vg_paod_amp | History of Amputation for PAOD |
| vg_paod_amp_loc_ipsi | Location of amputation on ipsilateral side of symptoms |
| vg_paod_amp_loc_contra | Location of amputation on contralateral side of symptoms |
| vg_dvt | History of DVT |
| vg_aneu | History of Aortic Aneurysm |
| vg_thromb | History of cardial/free floating thrombus |
| med_ascal | Ascal use prior to occlusion |
| med_ascal_dose | Dose of ascal in mg |
| med_clopi | Clopidogrel use prior to occlusion |
| med_clopi_dose | Dose of clopidogrel in mg |
| med_tica | Ticagrelor use prior to occlusion |
| med_tica_dose | Dose of ticagrelor use |
| med_dipy | Dipyridamoleuse prior to occlusion |
| med_dipy_dose | Dose of dipyridamol |
| med_vitk | Vitamin K antagonist prior to occlusion |
| med_doac | DOAC use prior to occlusion |
| med_doac_dose | Dose of DOAC prior to occlusion |
| med_heparin | Heparin prior to thrombolysis |
| med_stat | Statin use |
| med_lld | Lipid lowering medication prior to occlusions |
| med_ht | Anti-hypertensive medication use |
| med_ace | ACE-inhibitor use |
| med_bb | Beta blocker use |
| med_ca | Calcium antagonist use |
| med_diur | Diuretic medication use |
| med_ang | Angiotensin II antagonist use |
| med_dm | Diabetic medication use |
| sympt_date | Date of first symptoms |
| sympt_side | Side of symptoms |
| sympt_pain | Location of pain |
| sympt_pain_rest | Pain in rest |
| sympt_pale | Pale limb |
| sympt_cr | Capillar refill (sec) |
| sympt_puls | Pulses palpable |
| sympt_paresth | Paraesthesia |
| sympt_paresis | Paresis |
| sympt_cold | Cold limb |
| rutherford | Rutherford stadium |
| radio1 | Type of radiology test |
| radio2 | Type of radiology test |
| radio3 | Type of radiology test |
| artery | Which artery is occluded (most proximal) |
| artery_bypass | Type of bypass (if occluded artery=bypass) |
| artery_stent | In stent occlusion? |
| lab_hb | Hemglobin level at admission |
| lab_ht | Hematocrit level at admission |
| lab_leu | Leucocyte level at admission |
| lab_thromb | Thrombocte level at admission |
| lab_kreat | Kreatinine level at admission |
| lab_egfr | eGFR level at admission |
| lab_na | Sodium level at admission |
| lab_k | Potassium level at admission |
| lab_ureum | Ureum level at admission |
| lab_crp | CRP level at admission |
| lab_glucose | Glucose level at admission |
| lab_hba1c | HbA1c level at admission |
| lab_tc | Total Cholesterol at admission |
| lab_tg | Triglycerides at admission |
| lab_ldl | LDL at admission |
| lab_hdl | HDL at admission |
| lab_pt | PT at admission |
| lab_aptt | aPTT at admission |
| lab_inr | INR at admission |
| lab_ddimer | D-Dimer at admission |
| lab_fib | Fibrinogen level at admission |
| thromb_date | Date and time of start thrombolysis |
| thromb_med | Medication type used for thrombolysis |
| sheath_loc | Location of sheath |
| pta | PTA performed |
| revasc_compl | Complication at start thrombolysis? |
| thrombectomy | Mechanical thrombectomy performed? |
| bolus | Bolus given? |
| bolus_dose | dose of bolus |
| catheter | Type of intra-arterial catheter |
| angio1_date | Date and time of angiogram 1 |
| angio1_revasc | revascularization performed at angio 1 |
| angio2_date | Date and time of angiogram 2 |
| angio2_revasc | revascularization performed at angio 2 |
| angio3_date | Date and time of angiogram 3 |
| angio3_revasc | revascularization performed at angio 3 |
| stop | Date and time thrombolysis was stopped |
| stop_reason | Reason to stop thrombolysis |
| closure_device | Closure device inserted |
| additional | Additional surgical therapy after thrombolysis (ipsilateral, within admission) |
| hb1 | Level of Hb1 |
| hb1_time | Date and time of Hb1 |
| hb2 | Level of Hb2 |
| hb2_time | Date and time of Hb2 |
| hb3 | Level of Hb3 |
| hb3_time | Date and time of Hb3 |
| hb4 | Level of Hb4 |
| hb4_time | Date and time of Hb4 |
| hb5 | Level of Hb5 |
| hb5_time | Date and time of Hb5 |
| hb6 | Level of Hb6 |
| hb6_time | Date and time of Hb6 |
| hb7 | Level of Hb7 |
| hb7_time | Date and time of Hb7 |
| hb8 | Level of Hb8 |
| hb8_time | Date and time of Hb8 |
| hb9 | Level of Hb9 |
| hb9_time | Date and time of Hb9 |
| hb10 | Level of Hb10 |
| hb10_time | Date and time of Hb10 |
| packed_cell | Number of packed cells given |
| bleeding | Bleeding complication? |
| bleeding_stop | Has thrombolytic therapy stopped because of bleeding? |
| bleeding_loc1 | Location of bleeding 1 |
| bleeding_loc2 | Location of bleeding 2 |
| bleeding_hemodynamic | Hemodynamic status in bleeding |
| bleeding_action | Action on bleeding |
| bleeding_device | Bleeding due to closure device failure? |
| bleeding_trauma | Bleeding due to trauma during thrombolysis? |
| bleeding_major | Major bleeding? |
| trash | Trash for which continued thrombolysis or amputation |
| amputation | Amputation as complication of thrombolysis |
| reperfusion | Reperfusion syndrome with fasciotomy |
| death | Death during hospital stay |
| med_postac | Prescribed Anti coagulant after admission |
| readmission | Readmission within 30 days |
| reintervention30 | Reintervention within 30 days |
| reintervention90 | Reintervention within 90 days |
| death_date | Date of death |
