# Supplementary material for: Predictors of bleeding complications during catHeter-dirEcted thrombolysis for peripheral arterial occlusions (POCHET)
Source: PLoS One. 2024 May 9;19(5):e0302830. doi: 10.1371/journal.pone.0302830 (PMC11081216; doi:10.1371/journal.pone.0302830)
Supplement: Attachment — Submitted filename: 20240314 Response to Reviewers.docx [file pone.0302830.s004.docx]

**Response to Reviewers**

Journal Requirements:

When submitting your revision, we need you to address these additional requirements.

1. Please ensure that your manuscript meets PLOS ONE's style requirements, including those for file naming. The PLOS ONE style templates can be found at .

<https://journals.plos.org/plosone/s/file?id=wjVg/PLOSOne_formatting_sample_main_body.pdf> and 
<https://journals.plos.org/plosone/s/file?id=ba62/PLOSOne_formatting_sample_title_authors_affiliations.pdf>

*The manuscript has been revised to the PLOS ONE’s style requirements*

2. Please include “Protocol” in the manuscript  title.

“Protocol” has been added to the manuscript title

3. Please include a copy of the ethics committee approval, which should include the date of approval, the approval number, and signature or stamp from the ethics committee, and English translation, as an "Other" file.
In the Methods section of your revised manuscript, please include the full name of the institutional review board or ethics committee that approved the protocol, the approval or permit number that was issued, and the date that approval was granted.

The original letter of approval of the Biobank Research Ethics Committee and it’s English translation has been included as an “other” file with the latest submission.

Under the “ethical approval” header within the “Methods and Design” section, the approval number and the date of approval is added.

4. One of the noted authors is a group or consortium: The POCHET study group

In addition to naming the author group, please list the individual authors and affiliations within this group in the acknowledgments section of your manuscript. Please also indicate clearly a lead author for this group along with a contact email address.

The affiliation of the members of the POCHET study group have been added in the acknowledgment section. Also the lead author of the POCHET study group is indicated with a contact email address.

**Additional Editor Comments:**

I read the manuscript on this important topic with interest. I think that the study protocol is generally well written and will provide important contributions to those interested in the subject when the study is completed. The manuscript has been reviewed by three reviewers and their concerns are as follows. Please edit your manuscript according to the reviewers' suggestions and provide point-by-point responses. In particular, please provide the ethics committee approval of the study and the clinical trials register.


[Note: HTML markup is below. Please do not edit.]

Reviewers' comments:

Reviewer's Responses to Questions

**Comments to the Author**

1. Does the manuscript provide a valid rationale for the proposed study, with clearly identified and justified research questions?

The research question outlined is expected to address a valid academic problem or topic and contribute to the base of knowledge in the field.

Reviewer #1: No

Reviewer #2: Yes

Reviewer #3: Yes

2. Is the protocol technically sound and planned in a manner that will lead to a meaningful outcome and allow testing the stated hypotheses?

The manuscript should describe the methods in sufficient detail to prevent undisclosed flexibility in the experimental procedure or analysis pipeline, including sufficient outcome-neutral conditions (e.g. necessary controls, absence of floor or ceiling effects) to test the proposed hypotheses and a statistical power analysis where applicable. As there may be aspects of the methodology and analysis which can only be refined once the work is undertaken, authors should outline potential assumptions and explicitly describe what aspects of the proposed analyses, if any, are exploratory.

Reviewer #1: No

Reviewer #2: Yes

Reviewer #3: Yes

3. Is the methodology feasible and described in sufficient detail to allow the work to be replicable?


Descriptions of methods and materials in the protocol should be reported in sufficient detail for another researcher to reproduce all experiments and analyses. The protocol should describe the appropriate controls, sample size calculations, and replication needed to ensure that the data are robust and reproducible.

Reviewer #1: No

Reviewer #2: Yes

Reviewer #3: Yes

4. Have the authors described where all data underlying the findings will be made available when the study is complete?

The [PLOS Data policy](https://eur05.safelinks.protection.outlook.com/?url=https%3A%2F%2Fjournals.plos.org%2Fplosone%2Fs%2Fmaterials-and-software-sharing&data=05%7C02%7CB.M.Mol%40umcutrecht.nl%7Cffa75306f7064364de0a08dc1c63c474%7Cdcdf4a3dd0c04a6394cf781981249be5%7C0%7C0%7C638416463174539310%7CUnknown%7CTWFpbGZsb3d8eyJWIjoiMC4wLjAwMDAiLCJQIjoiV2luMzIiLCJBTiI6Ik1haWwiLCJXVCI6Mn0%3D%7C3000%7C%7C%7C&sdata=T5mNpQ1brzQzXP0R9XkcxvKiSfRqC6axwMVbRrWJlRo%3D&reserved=0) requires authors to make all data underlying the findings described in their manuscript fully available without restriction, with rare exception, at the time of publication. The data should be provided as part of the manuscript or its supporting information, or deposited to a public repository. For example, in addition to summary statistics, the data points behind means, medians and variance measures should be available. If there are restrictions on publicly sharing data—e.g. participant privacy or use of data from a third party—those must be specified.

Reviewer #1: No

Reviewer #2: Yes

Reviewer #3: No

5. Is the manuscript presented in an intelligible fashion and written in standard English?

PLOS ONE does not copyedit accepted manuscripts, so the language in submitted articles must be clear, correct, and unambiguous. Any typographical or grammatical errors should be corrected at revision, so please note any specific errors here.

Reviewer #1: Yes

Reviewer #2: Yes

Reviewer #3: Yes

6. Review Comments to the Author

Please use the space provided to explain your answers to the questions above and, if applicable, provide comments about issues authors must address before this protocol can be accepted for publication. You may also include additional comments for the author, including concerns about research or publication ethics.

You may also provide optional suggestions and comments to authors that they might find helpful in planning their study.


(Please upload your review as an attachment if it exceeds 20,000 characters)

Reviewer #1: Overall evaluation: The order of work in this study protocol should be reversed. First full ethical approval, then Clinical Trials registration, then submission of study protocol including statements of full ethical approval and Clinical trial registration number.
It should be clear from the protocol that some data will be collected and interpreted as part of the emergency intervention for proper patient care while some blood samples will be collected for “the future”.
One large problem you will encounter is that most bleeding events, also major, is related to access-site related bleeding that has nothing to do with anticoagulation, coagulation or fibrinolytic disturbances. Have you thought of that? Your primary endpoint should be major non-introducer-related major bleeding, and your estimated sample size should be based on assumption upon this endpoint, and not all major bleeding complications.

Dear reviewer,

The authors would like to thank you for your elaborate evaluation of the study and your very thorough comments. We adjusted the manuscript on these topics and clarify why we made certain decisions. Before we answer the list below, let us please answer your general comments.

- The nature of this study is observational and does not assign patients to one or different interventions which are compared between groups. For this study, all included patients are treated according to the predefined and standardized treatment protocol. The assessment of the best type of thrombolysis protocol falls beyond the scope of this study. Also because there are many variables to be studied to define the optimal protocol i.e. type of thrombolysis; dosage; access routing; duration of therapy, additional heparinization; interval of control angio etc etc.
- Donated blood samples are stored in the biobank for future analysis. Based on these future analysis, a new study can be conducted which can analyse possible new biomarkers that predict bleeding complications during thrombolysis. Because of the observational setting of the study, our study is not a clinical trial as defined by the International Committee of Medical Journal Editors (ICMJE) as there is no intervention. The definitions states:

  “The ICMJE defines a clinical trial as any research project that prospectively assigns people or a group of people to an intervention, with or without concurrent comparison or control groups, to study the relationship between a health-related intervention and a health outcome”

  <https://www.icmje.org/recommendations/browse/publishing-and-editorial-issues/clinical-trial-registration.html>

For this, the authors believe that a registration as a clinical trial is not applicable. To clarify this, we added “ As the nature of this study is an observational biobank study, registration as a clinical trial is not applicable“ to the study design section in line 120-121 ( the clean revised manuscript.

- Reviewer #1 stated that the order of work in this study protocol should be reversed, but since the registration as a clinical trial is not applicable, it is of our believe that the suggested order is no longer applicable. To clarify this, we added “ As the nature of this study is an observational biobank study, registration as a clinical trial is not applicable“ to the study design section in line 120-121 of the clean revised manuscript.
- The suggestion about the major bleeding complications definition is a very important recommendation. There is a very significant difference between access site related bleedings and any other bleeding during catheter direct thrombolysis. This difference was not clearly stated in the manuscript and therefore, the definition of the primary endpoint is edited to “non- access site related major bleeding” in line 165 (clean revised manuscript) in the endpoint definition section and in line 40 (clean revised manuscript) of the abstract.

Specific comments:
1. Abstract. Add how many centers. We thank the reviewer for this important comment because the number of including centers is important information. The reason we have not included the number of including centers is because the possibility for other centers to join later on in including patients for the POCHET trial. By stating the number of including centers at this point in time, this information will be incorrect when other authors will reference to this protocol paper. It is of course very important information to state the number of including centers when the results of the POCHET biobank are published. Currently, we have two centers including patients and expect more centers to include patients in the year 2024.

2. Add Clinical Trials registration number. See first bullet point above.

3. Abstract – define major bleeding already here. As mentioned above, the primary endpoint has been changed in the abstract as ‘non-access related major bleeding” as mentioned above.

4. The full ethical approval of this study is missing and should be included before the study protocol is submitted to a journal. In this ethical approval, the maximal amount of extra blood taken from the patient outside of clinical routine should be stated, and a reference given that it is ok to withdraw a certain amount of blood without any clinical consequences. The comment is on the ethical approval is a very significant recommendation. This study was approved by the Biobank Research Ethics Committee of the University Medical Center Utrecht, which is the central organ for reviewing new biobank protocols. It’s approval letter of the biobank protocol, including English translation, is submitted with this revised manuscript. In this biobank protocol it is stated that during thrombolysis, along with general blood collections every 6 hours, blood collections for the biobank were performed, consisting of a 6 ml EDTA and a 4,5ml citrate tube. With a maximum duration of thrombolysis of 72 hours and an extra donation to the biobank before thrombolysis would start, the maximum blood collections for the biobank per patient will add up to a maximum of 13 separate donations of 10,5 ml per donation. Blood donations to the biobank would stop when thrombolysis stops, which means that in general practice, a lot of patients will donate less than 13 times. This is further clarified in the Method section under sub header “Study design” and “lab manual for blood samples” in lines 122 (clean revised manuscript) and 198 (clean revised manuscript) and stated below.

Line 122: “With a maximum CDT-duration of 72 hours, the number of blood collections are maximised at 13 times”
Line 198: “When CDT stops, blood collections for the biobank will stop immediately.”

A total maximum of 136,5 ml per patient is taken. Amrein et al (doi 10.1016/j.blre.2011.09.003) reviewed the risks of blood donation, which were mainly vena puncture related or had to do with vasovagal collapses. For this study, these risks are not applicable, as blood samples are collected by a venous catheter. Long term risks of blood donation would mainly be iron deficiency syndrome, which was more seen in people who donated blood more frequently. The patients in our study, only donate blood to the biobank during thrombolysis treatment and the total amount of blood taken is only one third when compared to a regular blood donation (max 136,5ml vs 500 ml). This contributes to the safety of the blood donation for the POCHET biobank, which is very important. This is why it is further elaborated in the manuscript (line 126 of clean revised manuscript, “donating a maximum of 136.5 ml blood to the biobank is considered safe and does not affect standard healthcare”.) and in this letter.

5. Some kind of power calculation would benefit this study protocol. How many patients would be needed to have some clue whether fibrinogen is a marker of major bleeding?

Thank you for this important recommendation. A power calculation in clinical research aids researchers in the risk of under or over inclusion. However, the POCHET study is not a clinical study, but a biobank. As previous studies mostly had a retrospective analysis as design, there is a broad range of expected endpoints, which also differed in their definitions amongst studies. As such, the prospective POCHET registry using strict defined endpoints and standardized protocol will help to set the new outcome parameters which can subsequently be used to perform power calculations for future thrombolysis based studies.

The most important value of a biobank is that it holds tissue or blood for future research for all kinds of research questions. Every research question can have its own effect and thereby have its own power calculation. This is further elaborated in the subheader “Sample size” in the “Methods and design” section.

6. Data collection. The clinical biochemistry assays for example for fibrinogen may differ across centers. Have you checked that? How do you intend to calibrate between assays?

This is a very important remark. All plasma samples will be stored temporarily in the participating centers at -80° Celsius. No biochemistry assay is being done in different centers. Samples are transported on dry ice every few months to the central biobank in Utrecht where they again will be stored at -80° Celsius. All biochemistry assays will then be done in a single laboratory. This is further elaborated in the subheader “Lab manual for blood samples” in the “methods and design “ section in line 194 of the clean revised manuscript and stated below.

“All future analysis will be done in a single laboratory facility,”

7. Data collection. No use of for instance REDCap data management system?

Thank you for this comment. Although it is stated that the data is securely stored, the way it is stored is not mentioned in the manuscript. This is changed in the revised manuscript in line 203 of the clean revised manuscript and stated below

“Data regarding patient characteristics at presentation such as comorbidities, medication use, occlusion specifics and data of hospital admission and follow-up will all be collected and stored in the data management system Castor EDC”

8. Definition of major bleeding – you should add necessity of transfusion of at least 2 units of blood during the in-hospital stay. Give reference: Schulman et al. J Thromb Haemostat 2010; 8: 202-204. Even though you argue against this, it is clinically relevant since many patients with ALI also have concomitant anemia at admission (around 30%).

The reviewer has a very relevant comment on the addition of at least 2 units of blood during the in-hospital stay and this was extensively discussed within the adjudication committee. The conclusion of this discussion was that although it is clinically relevant whether patients need two units of blood, there is a big difference between patients that are admitted with anemia and patients that are not. Patients with anemia and an otherwise minor bleeding would need a blood transfusion sooner than the same patients admitted with normal hemoglobin levels. Especially because hemoglobin levels drop during admission even when no bleeding occurs. Thereby, major bleeding complication rates would be unnecessarily overestimated.

Secondly, as Schulman stated (DOI: [doi.org/10.1111/j.1538-7836.2009.03678.x](https://doi.org/10.1111/j.1538-7836.2009.03678.x)), incorporation of criteria for surgical blood loss, such as transfusion of more than two units of red blood cells, does not support the clinical concept of major blood loss in surgery patients, as this is linked to the type of surgery or intervention. A criterion covering events of excessive and unexpected blood loss are marked with the type of intervention that was implemented. That is why these events were incorporated in the definition of major bleeding complication for the POCHET trial, such as re-intervention.

Nevertheless, blood transfusion in relation to any bleeding complication is very relevant information and of course will be collected. Also because with this information, different bleeding complication classifications can be deducted from the data and compared.

This points were further clarified in line 269 - 278 (clean revised manuscript) of the discussion section and stated below:

“Furthermore, for the definition of the primary endpoint of this study, non-access site related major bleeding complication, the commonly used criterion “necessity of transfusion of 2 or more units of blood” is intentionally not used as it is highly linked to the type of surgery or intervention in the surgical population (17). A criterion covering events of excessive and unexpected blood loss are marked with the type of intervention that was implemented. That is why these events were incorporated in the definition of major bleeding complication for the POCHET trial, such as re-intervention. Nevertheless, blood transfusion in relation to any bleeding complication is relevant information and will be collected . With this information, different bleeding classifications can then be analysed for which classification is most clinically relevant for the population of patients receiving CDT.”

9. Statistics. Can you expand on how you will be able to correct for competing interest? I mean if a patient dies, there will be no possibility of major amputation.

Competing events are very relevant and we thank the reviewer for mentioning the lack of naming it in our manuscript. An extra secondary endpoint is added: non-bleeding related death. Because, as the reviewer so rightly states, when a patient dies of a non-bleeding related death, there will be no risk of major amputation and therefore this competing event should be included as a secondary endpoint.

10. Limb salvage. Do you mean major amputation? Please define.

The authors agree that the definition of limb salvage is not clear. Therefore, we changed it in the manuscript into major amputation, being above the ankle amputation

11. Ref 12 and 15 are the same.
With shame I note this sloppy error and it has been corrected in the revised manuscript.

Reviewer #2: The present study protocol aims to investigate the possible biomarkers to identify patients with high bleeding risk associated with catheter directed thrombolysis. The topic is clinically relevant although the multiple treatment protocols add to the challenge of clinical trial. The use of either Alteplase or Urokinase would make study more standardized. However if target 500 patients will be reached the post hoc comparation between regimen and biomarkers might be possible.

We are very grateful that you have reviewed our manuscript and that you have provided new insights and additions to it. We are happy to answer your questions and improve the manuscript based on your advice. To reply on the comment about the comparison between different thrombolytic medications: the standard protocol, which is added to the manuscript as a supplement, dictates the use of alteplase or urokinase. When the data are analysed, the results will be corrected for the specific medication that is used.

Only some minor notes on the manuscript:

Since present study is a clinical trial it would be logical to be registered as clinical trial at for example web registry ClinicalTrials.gov and registry number to be added to the manuscript.

The nature of this study is observational and does not assign patients to one or different interventions which are compared between groups. For this study, included patients are treated according to a standard treatment protocol. The best type of thrombolysis protocol falls beyond the scope of this study. Donated blood samples are stored in the biobank for future analysis. Based on these future analysis, a new study can be conducted which can analyse possible new biomarkers that predict bleeding complications during thrombolysis. Because of the observational setting of the study, the study is not a clinical trial as defined by the International Committee of Medical Journal Editors (ICMJE) as there is no intervention. The definitions states:

“The ICMJE defines a clinical trial as any research project that prospectively assigns people or a group of people to an intervention, with or without concurrent comparison or control groups, to study the relationship between a health-related intervention and a health outcome”

<https://www.icmje.org/recommendations/browse/publishing-and-editorial-issues/clinical-trial-registration.html>

This is why it is of the authors believe that a registration as a clinical trial is not applicable.

Study design it would be more informative to add a nice flow chart. The reference to S1 is ok, but nice flow chart would make manuscript more interesting. Also figures 1-4 are nice but flow chart would be more accurate and present figures could support the flow chart?

This is a very nice suggestion of the reviewer, so we have added a flowchart as a summary of the POCHET trial as figure 4, which is referred to in line 207 (clean revised manuscript) and added below.

Allergy against Alteplase or Urokinase not contraindication according S1? Please check absolute and relative contraindications.

We thank the reviewer for finding this error in the supplemental material. Although we have been working with the contra-indication “allergy to thrombolytic medication”, somehow this must have slipped out of the manuscript. This error has been accounted for in the revised manuscript and supplemental material.

Figure 3 one might get impression that the blood samples are collected from introduction sheet close to thrombus, which might even be interesting addition considering the aim of the study?

The thought of collecting blood samples from the sheath has crossed the minds of the research team. However, we came to the conclusion that venous blood samples would be more accurate, as thrombolytic medication or heparin might stick to the sheath, transfers into the blood sample and interferes with the biomarkers that are related to coagulation and hemostasis. This was further elaborated in the “lab manual for blood samples” subsection of the methods and design section in line 188-190 (clean revised manuscript) and stated below:

“Blood samples are not collected by using the sheath or thrombolysis catheter, as thrombolytic medication might interfere with the coagulation biomarkers used for future analysis.”

Biobank approval number available? If it would be nice to add it to the text.

This study was approved by the Biobank Research Ethics Committee of the University Medical Center Utrecht, which is the central organ for reviewing new biobank protocols. It’s approval letter of the biobank protocol, including English translation, is submitted with this revised manuscript. The approval number of the Biobank Research Ethics Committee is added in the manuscript.

Reviewer #3: Dear authors

Thanks for sharing your protocol. I have some few suggestions/doubts that may improve your data.

We are delighted with all the advice and recommendations of reviewer #3 and look forward to implement his/her suggestions to improve the manuscript.

1)Title and abstract are interesting and highlight main points of the study. I think abstract must draw attention to the readers. I suggest report which biomarkers will be included at your research at the abstract.

We thank the reviewer with the compliments on the title and abstract. In the Methods section in the abstract, several biomarkers are mentioned which have the special interest of the authors.

2) Inclusion criteria: How severe were the included patients related to the limb perfusion? Only ALI Rutherford I and IIa patients were included? Please, answer - You must be very clear as this is a very crucial feature to take in accout. Explain

We thank the reviewer for this comment, as the inclusion and exclusion criteria might not seem clear. The inclusion and exclusion criteria which are stated in the Methods and Design section are specifically intended for the POCHET study and should not be confused with thrombolytic treatment as a whole. The first step is for the treating physician to decide whether the acute limb ischemia can be treated with catheter directed thrombolysis. This could be Rutherford I up to IIb. Contra-indications for thrombolytic therapy are stated in the supplemental S1. Then, every patient that has an indication for catheter directed thrombolysis of the lower limb, can be included in the POCHET study, as long as the other POCHET specific in- and exclusion criteria are met. Obviously, the Rutherford classification for acute limb ischemia is part of the baseline characteristics so that it can be used as a possible confounder for bleeding complications.

This is further clarified under the subheader In- and Exclusion criteria in line 134 (clean revised manuscript and stated below:

“When the treating physician decides CDT is the treatment of choice for ALI, patients must fulfil the following inclusion criteria for the POCHET study:

- Subacute (<6 weeks) thrombotic occlusion of a native artery, bypass or stent in the iliac or femorodistal artery.
- 18 years of age.
- Written informed consent.

**Exclusion criteria**

A patient is not able to participate in this biobank study if one of the following criteria is present:

- No treatment indication for CDT
- Not willing to or not able to provide written informed consent
- Absolute or relative contra-indication for thrombolytic therapy (see Supplement 1)”

3) Exclusion criteria: IIB and II Rutherford ALI categories. Yes or not? Please, explain

This question is in line with your previous question (number 2): at first the treating physician must decide whether acute limb ischemia can be treated with catheter directed thrombolysis. The in- and exclusions criteria for thrombolytic therapy are stated in supplemental S1. When patients have a treatment indication for thrombolytic therapy, they can be included in the POCHET study as long as they do NOT meet one of the exclusion criteria which are stated in the Methods and Design section.

4) About potential biomarkers: I suggest you describe how they will be measured at blood samples - your protocol and findings must be reproductible for other groups , mainly anti-plasmin, plasminogen activator inhibitor and VES.

The method of measuring biomarkers is indeed a key aspect of the research that can be conducted after establishment of the POCHET biobank. The nature of this manuscript and protocol, as being a biobank, is not primarily on which potential new biomarkers can be measured in the blood that is collected, but states the design of how to biobank study is conducted. Several potential biomarkers are mentioned as an example but it goes beyond the scope of this manuscript to add the methods for such markers. After inclusion of enough patients, research questions and biomarkers questions can be answered using the biobank samples with precise and reproducible protocols for biomarker analyses.

5) Will fibinolytic agent be standartized? Is alteplase? Or other agents will be allowed? Please , be as clearer as possible.

We thank the reviewer for this important comment. Although literature suggests otherwise, in theory might different fibrinolytic medication lead to different bleedings risks. The standard protocol for thrombolytic treatment, which is added to the manuscript as a supplement, dictates the use of alteplase or urokinase, as its effect and risk are similar (DOI: [10.1002/14651858.CD001099.pub3](https://doi.org/10.1002/14651858.cd001099.pub3)). When the data is analysed, the results will be corrected for the specific medication that is used.

To further clarify this, we added this information in lline 114-116 (clean revised manuscript) and is stated below:

“Patients are treated with either Urokinase or Alteplase, as effects and risks are similar (10). Results will be corrected for thrombolytic medication given.”

6) Both ischemic upper and lower limbs will be included? Pleases, specify it.

As is stated in the inclusion criteria, only patients with acute limb ischemia of the iliac or femorodistal artery can be included in the POCHET study.

7) I suggest define 02(two) groups of endopoints: efficacy endpoints and safety endpoints (besides bleeding complications, I suggest reporting "limb salvage"). All your variables MUST be reported.

We are thankful for the reviewers comment on reporting all the variables and endpoint definitions. We have added a list of both in the supplemental material and are referring to this list in the revised manuscript in line 172 and 206 (clean revised manuscript) as stated below:
“A list of all endpoint definitions is available in the supplementals (S2).”
“A list of all included variables is available in the supplementals (S3).”

8) Is your protocol registered (or being registered) on a platform like clinical trials.gov?

The nature of this study is mostly observational and does not assign included patients to one or different interventions which are compared between groups. For this study, included patients are treated according to a standard treatment protocol. The best type of thrombolysis protocol falls beyond the scope of this study. Donated blood samples are stored in the biobank for future analysis. Based on these future analysis, a new study can be conducted which can analyse possible new biomarkers that predict bleeding complications during thrombolysis. Because of the observational setting of the study, the study is not a clinical trial as defined by the International Committee of Medical Journal Editors (ICMJE) as there is no intervention. The definitions states:

“The ICMJE defines a clinical trial as any research project that prospectively assigns people or a group of people to an intervention, with or without concurrent comparison or control groups, to study the relationship between a health-related intervention and a health outcome”

<https://www.icmje.org/recommendations/browse/publishing-and-editorial-issues/clinical-trial-registration.html>

This is why it is of the authors believe that a registration as a clinical trial is not applicable.

Please pay attention, mainly at question 2. It is very important to define your inclusion (and exclusion) criteria - it may be a potential source of bias

7. PLOS authors have the option to publish the peer review history of their article ([what does this mean?](https://eur05.safelinks.protection.outlook.com/?url=https%3A%2F%2Fjournals.plos.org%2Fplosone%2Fs%2Feditorial-and-peer-review-process%23loc-peer-review-history&data=05%7C02%7CB.M.Mol%40umcutrecht.nl%7Cffa75306f7064364de0a08dc1c63c474%7Cdcdf4a3dd0c04a6394cf781981249be5%7C0%7C0%7C638416463174546281%7CUnknown%7CTWFpbGZsb3d8eyJWIjoiMC4wLjAwMDAiLCJQIjoiV2luMzIiLCJBTiI6Ik1haWwiLCJXVCI6Mn0%3D%7C3000%7C%7C%7C&sdata=rDupz8kWWWDfPa6pX98yv6Hg5O811ygDdovG8uV55YA%3D&reserved=0)). If published, this will include your full peer review and any attached files.


If you choose “no”, your identity will remain anonymous but your review may still be made public.


**Do you want your identity to be public for this peer review?** For information about this choice, including consent withdrawal, please see our [Privacy Policy](https://eur05.safelinks.protection.outlook.com/?url=https%3A%2F%2Fwww.plos.org%2Fprivacy-policy&data=05%7C02%7CB.M.Mol%40umcutrecht.nl%7Cffa75306f7064364de0a08dc1c63c474%7Cdcdf4a3dd0c04a6394cf781981249be5%7C0%7C0%7C638416463174553402%7CUnknown%7CTWFpbGZsb3d8eyJWIjoiMC4wLjAwMDAiLCJQIjoiV2luMzIiLCJBTiI6Ik1haWwiLCJXVCI6Mn0%3D%7C3000%7C%7C%7C&sdata=hqn626fIL%2FF2FgLVG7NqbGrOUEayvlFeKwZWuZ7tAMY%3D&reserved=0).

Reviewer #1: No

Reviewer #2: No

Reviewer #3: No
